# Association of depression with newly diagnosed type 2 diabetes among adults aged between 25 to 60 years in Karachi, Pakistan

**DOI:** 10.1186/1758-5996-2-17

**Published:** 2010-03-19

**Authors:** Shazia Perveen, Muhammad S Otho, Muhammad N Siddiqi, Juanita Hatcher, Ghazala Rafique

**Affiliations:** 1 Cardiothoracic Section, Department of Surgery, Aga Khan University Hospital, Stadium Road, PO Box 3500, Karachi 74800, Pakistan; 2 Global Funds for AIDS, TB and Malaria, Round 7, Directorate of Malaria, 1st Floor, Allergy Center Chak Shehzad, Islamabad; 3 Department of Psychiatry, Aga Khan University, Karachi, Pakistan; 4 Department of Community Health Sciences, Aga Khan University, Karachi, Pakistan; 5 Epidemiology and Biostatistics Division, Department of Community Health Sciences, Aga Khan University, Karachi, Pakistan

## Abstract

**Background:**

The combination of depression with type 2 diabetes is a public health problem. If diabetes is managed in its initial phase, the morbidity and mortality due to this combination may be prevented at an early stage. Therefore, we aimed to determine the association of depression with newly diagnosed type 2 diabetes among adults aged between 25 to 60 years in Karachi, Pakistan.

**Methods:**

From July 2006 to September 2007, a matched case control study (n = 592) was conducted in Civil Hospital, Karachi. Incident cases of type 2 diabetes (n = 296) diagnosed within one month were recruited from diabetic Out Patient Department (OPD) of Civil Hospital, Karachi. They were matched on age and sex with controls (n = 296), who were attendants sitting in the medical out patient department of the same hospital, recruited on the basis of absence of classical symptoms of polyuria and polydispia along with random blood glucose level of <200 mg/dl measured by a glucometer. Depression was identified by the Siddiqui Shah Depression Scale. Conditional logistic regression was applied to examine the association of depression and other independent variables with newly diagnosed type 2 diabetes at 95% C.I. and P < 0.05.

**Results:**

The study comprised of 592 subjects with 432(73%) males and 160(27%) females. Depression was significantly associated with newly diagnosed type 2 diabetes having mild level (mOR: 3.86; 95%CI: 2.22,6.71) and moderate to severe level (mOR: 3.41; 95%CI: 2.07,5.61). History of (h/o) gestational diabetes (mOR: 2.83; 95%CI: 1.05,7.64), family h/o diabetes (mOR: 1.59; 95%CI: 1.04,2.43), nuclear family (mOR: 1.75; 95%CI: 1.14,2.69), BMI (mOR: 1.62; 95%CI: 1.01,2.60 for obese and mOR: 2.12; 95%CI: 1.19,3.79 for overweight vs healthy to underweight) were also significantly associated with outcome, adjusting for age, sex, marital status, h/o smoking and h/o high BP.

**Conclusions:**

Diabetics should be screened simultaneously for depression and concomitant preventive strategies for gestational diabetes, nuclear family and high BMI should also be used to prevent mortality/morbidity among patients between 25 to 60 years of age.

## Background

Type 2 diabetes and depression are two long course diseases with modifiable risk factors. Diabetes, the fourth leading cause of death has affected an estimated 246 million people in the world [1]. In 2007, the number of people affected by diabetes in Asia rose to 110 million, with different proportion for young and middle aged people [2]. Among type 2 diabetics, the prevalence of newly diagnosed diabetes in people aged 25 years or above is 5.1% males and 6.8% females in urban areas and 5% males and 4.8% females in rural areas of Pakistan [3]. Depression accounted for 10% prevalence in primary care settings at various developed as well as developing countries [4]. In Pakistan, mean overall prevalence of anxiety and depressive disorders is 34% [5].

Among patients with type 2 diabetes, depression is found to be strongly associated with increased morbidity and mortality [6,7]. Multiple studies from developed countries have proved positive association of type 2 diabetes with depression [8,9]. Similarly, in the developing countries of South Asia, the association was significant in rural areas of Bangladesh, predominantly among females [10]. Zhao et al found this association in young adults [11] and it has also been found to be significantly associated with physical inactivity [12]. Beside age and gender [13], multiple other risk factors and co-morbidities are associated with either one or both diseases. These include socioeconomic status [14], past history of (h/o) depression [15], family h/o diabetes [16], h/o gestational diabetes [17], family type [18], Body Mass Index (BMI) [19-21], h/o smoking [22], h/o high cholesterol [23,24] and h/o high blood pressure (BP) [25].

Epidemiological transition and lifestyle modification in South Asian countries have increased the prevalence of type 2 diabetes and depression. The healthcare systems of these countries have adequate services for diagnosis and management of type 2 diabetes but depression is not considered to be a regular or essential part of health or social milieu. Going to a psychiatrist for any mental problem including depression is regarded as a taboo. Usually people follow cultural or religious traditions to address mental illnesses rather than getting professional help. Untreated depression might be responsible for increase in diabetic co-morbidities because of poor self care, non-compliance with diet and exercise guidelines or lapse in filling prescription for oral diabetes medication [19]. Eventually, positive association between diabetes and depression has been identified [10]. Exploring this association at the initial stage of type 2 diabetes would help clinicians and psychiatrists to find avenues for preventive strategies and advocacy could be done at policy making level for early reduction of morbidity and mortality.

Although developed countries have enough evidence on this fatal combination, developing countries still have insufficient data on association of depression with type 2 diabetes especially with newly diagnosed type 2 diabetes. We, therefore, explored the association of depression with newly diagnosed type 2 diabetes among adults aged between 25 to 60 years in Karachi, Pakistan.

## Research design, patients and methods

From July 2006 to September 2007, a matched case control study (n = 592) was conducted in the Out Patient Department (OPD) of Civil Hospital, Karachi. After being granted permission by competent authorities, incident cases of type 2 diabetes (n = 296) were identified opportunistically and recruited from diabetic OPD of Civil Hospital, Karachi. Cases were operationally defined according to American Diabetic Association guidelines [26] which requires the "presence of any two of the following: Symptoms of polyuria (frequent urination) and polydispia (increased thirst and consequent increased fluid intake) plus random blood glucose level > = 200 mg/dl (11.1 mmol/l), or elevated fasting blood glucose level > = 126 mg/dl (7.0 mmol/l) or 2 hour post prandial glucose level (> = 200 mg/dl (11.1 mmol/l) after 75 gm glucose load". Newly diagnosed type 2 diabetics were incident cases of type 2 diabetes diagnosed within the last one month. They had been referred to the OPD from wards or doctors of the same hospital or other hospitals of the city. The diagnosis made one month earlier was confirmed by documented proof in the form of diabetic card or prescription of medication or referral form. All those who failed to show proof of diagnosis of diabetes within the previous one month or could not give valid signed consent or refused to participate in the study or pregnant women were excluded from the study.

One control (n = 296) per case was recruited from medical OPD adjacent to diabetic OPD of the same hospital. They were the attendants accompanying medical patients waiting in medical OPD for their turn of clinical care. Those attendants who did not have polyuria and polydispia and had random blood glucose level of < 200 mg/dl (measured by glucometer) were selected as control. The attendants of cases under study, those having a blood relationship with respective cases or those who shared the same household environment with the case or had polyuria and polydispia or were detected as having random blood glucose level > = 200 mg/dl were excluded from the study. Matching on age and sex was done to minimize variation among cases and controls. Initially, on first two days of week, a bulk of 30 cases were identified and listed. On 3^rd ^day, eligible controls were matched on age and sex with the listed cases. From 4^th ^day onwards, cases and controls were recruited on alternate days to complete the sample size. Separate informed consents were used for cases and controls and study subjects were interviewed by two trained data collectors through pre-tested structured questionnaire for exploring the association of depression (level) and other hypothesized confounding factors with newly diagnosed type 2 diabetes.

The main Independent Variable, "depression" was screened through Siddiqui Shah Depression Scale (SSDI) Questionnaire. It was validated in Pakistani population for three different categories of depression: mild, moderate and severe [27]. In this study three categories of depression was used: no depression (score 0-25), mild depression (score 26-36) and moderate to severe depression (score 37+). For obesity and overweight, Body Mass Index (BMI) at the Asian cut off was measured (underweight: <18.50 kg/m^2^, healthy: 18.50 to 22.90 kg/m^2^, overweight: 23.00 to 24.90 kg/m^2^, obese: >25.00 kg/m^2^) [28] by height scale & calibrated bathroom scales. Information about socio-demographic factors such as age, sex, marital status, socioeconomic status, educational status, occupation and house ownership was gathered. Past history of depression, family h/o diabetes, h/o gestational diabetes, nuclear family type, h/o smoking, h/o exercise, h/o high cholesterol and h/o high BP, past h/o depression was collected by means of verbal inquiry from the study subjects.

Participation in the study was voluntary and the confidentiality of the study subjects was maintained. Potential patients of newly diagnosed type 2 diabetes identified in the control group or of depression detected in either cases or controls were given an authentic referral letter and sent to the OPD clinics of Civil Hospital, Karachi for timely management.

This study is a product of thesis done for Master in Epidemiology and Biostatistics at Aga Khan University Hospital, Karachi. It was ethically approval by Ethics Review Committee of Aga Khan University Hospital, Karachi (575 CHS/ERC-06) and was funded by Aga Khan University Research Council Grant (Project ID: 06GS008 CHS).

### Statistical analysis

Taking 15% (approximately) [29,30] as the prevalence of risk among controls (p_0 _= 0.15, α = 5% and β = 0.2 at 95% confidence level with corresponding odds ratio of 2 and inflating to 10% for the non-response and errors), sample size calculated by NCSS Pass statistical software was 592. Data was entered into EpiInfo version 6.04 and subsequently converted to SAS version 9.1 (Statistical Analysis Software Inc. NC USA) for performing univariate and multivariate analyses. Frequencies and percentages were computed for all the categorical variables. Newly diagnosed type 2 diabetes (present or absent) was taken as dependent variable. The category of independent variables with minimum level of association with newly diagnosed type 2 diabetes was taken as reference. Conditional logistic regression was performed to evaluate the association of depression and other independent variables with newly diagnosed type 2 diabetes at 95% C.I. and P < 0.05, controlling for potential confounders.

## Results

Table 1 shows that the study sample included 432(73%) male and 160(27%) females with a higher proportion of married ones (cases: 227(76.69%); controls: 251(84.80%). Although the proportion of working people in both cases and control was equal (cases: 59(19.93%); controls: 59(19.9%) but both mainly belonged to middle socioeconomic status (cases: 145(48.99%); controls: 165(55.74%). The proportion of past h/o depression in newly identified type 2 diabetics was higher in cases as compared to controls (cases: 49(16.55%); controls: 41(13.85%).

**Table 1:** Baseline characteristics of study subjects in cases & controls.

Male to female ratio is 432(73%): 160(27%)		
**Characteristics**	**Cases (n = 296)**	**Controls (n = 296)**
	n* (%)	n* (%)

**Marital status**		
- Married	227 (76.69%)	251 (84.80%)
**Family type**		
- Nuclear family	207 (70.61%)	175 (59.12%)
- Extended family	87 (29.39%)	121 (40.88%)
**Socioeconomic status**		
- Lowest	91 (30.74%)	85 (28.72%)
- Middle	145 (48.99%)	165 (55.74%)
- Upper	60 (20.27%)	46 (15.54%)
**Occupation**		
- Salaried workers	29 (9.8%)	23 (7.77%)
- Daily wages laborer	31 (10.47%)	36 (12.16%)
- Not working	236 (79.73%)	237(80.07%)
**House ownership**		
- Shared accommodation	15 (5.07%)	11 (3.72%)
- On rent	122 (41.21%)	114 (38.51%)
- Own house	159 (53.72%)	171 (57.77%)
**Illiterate**	150 (50.68%)	160 (54.05%)
**H/o smoking**	21 (7.09%)	20 (6.76%)
**H/o high cholesterol**	16 (5.41%)	12 (4.05%)
**H/o exercise**	51 (17.23%)	35 (11.83%)
**Past h/o depression**	49 (16.55%)	41 (13.85%)

* frequency

Univariate analyses calculated unadjusted odds ratio and 95% confidence interval at 25% significance level to include most of the biologically and statistically important independent variables (Table 2). Conditional logistic regression was applied and significant association of depression (level), history of (h/o) gestational diabetes, nuclear family type, family history of diabetes and BMI (obese vs healthy to underweight and overweight vs healthy to underweight) with newly diagnosed type 2 diabetes were found, independent of confounding effect of age, sex, marital status, h/o smoking and h/o high BP (Table 3). The odds of mild depression among cases were 3.86 times the odds among controls (95% CI: 2.22, 6.71) while odds of moderate to severe depression among cases were 3.41 times the odds among controls (95% CI: 2.07, 5.61). Cases were 2.83 times more likely to have history of gestational diabetes (95% CI: 1.05, 7.64), 1.79 times more likely to have nuclear family type (95% CI: 1.14, 2.69) and 1.59 times more likely to have family history of diabetes as compared to controls (95% CI: 1.04, 2.43). Also, BMI showed statistically significant association with newly diagnosed type 2 diabetes (obese: mOR = 1.62; 95% CI: 1.01, 2.60 and overweight: mOR = 2.12; 95% CI: 1.19, 3.79).

**Table 2:** Univariate aanalyses of associated factors with newly diagnosed type 2 diabetes (n = 592)

Characteristics	Cases (n = 296) n(%)	Controls (n = 296) n(%)	Unadjusted mOR^# ^(95% C.I.**)
**Depression level^+1^**			
- Moderate to severe	103 (34.80%)	63 (21.28%)	2.79 (1.84, 4.32)^++^
- Mild	73 (24.66%)	40 (13.51%)	3.13 (1.94, 5.06)^++^
**Marital status (single)^+2^**	69 (23.31%)	45 (15.20%)	1.77 (1.14, 2.76)^++^
**Nuclear family type^+3^**	209 (70.61%)	175 (59.12%)	1.71 (1.12, 2.44)^++^
**Family h/o diabetes^+4^**	106 (35.81%)	64 (21.62%)	1.93 (1.35, 2.77)^++^
**H/o high blood pressure^+5^**	195 (65.88%)	173 (58.45%)	1.42 (0.99, 2.03)^++^
**H/o gestational diabetes^+6^**	23 (7.77%)	6 (2.03%)	3.83 (1.56, 9.41)^++^
**BMI^+7^**			
- Obese	140 (47.30%)	150 (50.68%)	1.11 (0.76, 1.64)^++^
- Overweight	77 (26.01%)	52 (17.57%)	1.81 (1.12, 2.92)^++^

+ References: +1 no depression, +2 married, +3 extended family type, +4 no family history of diabetes, +5 no history of high BP, +6 no history of gestational diabetes, +7 healthy

++ Significant: at P < .25 # matched odds ratio ** Confidence Interval

**Table 3:** Conditional logistic regression analyses for factors associated with newly diagnosed type 2 diabetes (n = 592)

Characteristics	Adjusted mOR^# ^(95% C.I. **)
**Depression level^+1^**	
- Moderate to severe	3.41 (2.07, 5.61)^++^
- Mild	3.86 (2.22, 6.71)^++^
**H/o gestational diabetes^+6^**	2.83 (1.05, 7.64)^++^
**Nuclear family type^+3^**	1.75 (1.14, 2.69)^++^
**BMI^+7^**	
- Obese	1.62 (1.01, 2.60)^++^
- Overweight	2.12 (1.19, 3.79)^++^
**Family h/o diabetes^+4^**	1.59 (1.04, 2.43)^++^

+ References: +1 no depression, +6 no history of gestational diabetes, +3 extended family type, +7 healthy, +4 no family history of diabetes

++ Significant: at P < .05 # matched odds ratio ** Confidence Interval

Our study sample had 68(23%) of controls with impaired glucose tolerance (having random blood glucose 140 mg/dl to 199 mg/dl). To check the validity of final model on the whole study sample 592(100%), two more additional analyses were conducted: by excluding those with impaired glucose tolerance 456(77%) and on those who had impaired glucose tolerance 136(23%). All three analyses showed almost similar results. Therefore, we can conclude that depression is independently associated with newly diagnosed type 2 diabetes adjusting for potential confounders.

## Discussion

The study findings revealed that depression is significantly associated with newly diagnosed type 2 diabetes among adults aged 25 to 60 years. Type 2 diabetes needs adherence to long term treatment regimens including regular medical examination, investigating blood sugar levels and subsequent dietary, oral or injectable treatment. During its course, fear of complications, feeling of helplessness and non-compliance to treatment may cause depression. Previously published literature proved that there is three times higher risk of depression among diabetics as compared to non-diabetics [11]. Their concomitant existence may adversely affect the working capacity of the individuals. Our results also show that mild depression has higher odds ratio as compared to moderate to severe depression (refer: Table 3). Most probably, diagnosis of type 2 diabetes might have led to mild depression which but later on association decreased due to patient's acceptance of the disease. Therefore, it is a strong point for policy makers to formulate preventive strategies against this fatal mix in the initial stage of diagnosis.

Along with lifestyle medication, family structure in Pakistan has been changed from extended to nuclear family type in Pakistan. Most of the families are living as nuclear families these days. It means parents live with their children only. Homes without grandparents might have reduced the burden of responsibilities and cumulative support could be rendered for prevention and management of type 2 diabetes with co-morbidities and complications. Our findings strongly support the association of nuclear family with newly diagnosed type 2 diabetes. It is consistent with previous literature [31] but Irving et al. [32] showed insignificant association with family structure which is needed to be explored with longitudinal study design. Along with lifestyle modification, genetic factors have also been documented as an associated factor for type 2 diabetes. Framingham offspring study proved family history as a significant factor for type 2 diabetes [33]. Our study also reaffirmed the positive association of family history of diabetes [34]. Additionally, our results also re-confirmed the significant association of past history of gestational diabetes with type 2 diabetes [23,35]. It emphasizes the importance of selective screening for gestational Diabetes.

Our study results show (refer to Table 1) that cases of diabetes were exercising more than control but it might be due to the prompt compliance of the advice given by their doctors after diagnosis of diabetes. But with the passage of time and progression of diabetes, patients might have ignored the exercise routine. Supporting the fact, newly diagnosed type 2 Diabetics have higher BMI as overweight and obese; which might be due to non-exercise culture or ineffective health seeking behavior.

One of the major strength of the study is its matched case control design which controlled two fundamental confounders of age and sex at design stage. Selection timings of both cases and controls were the same and they were interviewed under strictly similar conditions using the same questionnaire. Recruitment of cases and controls was done on the basis of disease status which avoided selection biases from the study. Both cases and controls were selected from the same source population, so they were representative of the source population with respect to depression within age sex matched strata. It achieved the goal of unbiased control. The screening tool used for depression exclusively screened out depression, thus avoided false positive results due to anxiety and somatic symptoms. Finally, sensitivity analysis was done by analyzing data set in different subgroups to address the sample size issue for wide range of prevalence. All showed no major differences among themselves.

Since depression is taken as a stigma, subjects suffering from depression might have refused to acknowledge it. Another limitation of the study is that controls were selected on the basis of Self-reported absence of symptoms and screening of random blood glucose by glucometer with only one reading which is against the established standard of diagnosis of type 2 diabetes [26].

It could not be verified by laboratory investigation because the study was the course requirement of a Master of Epidemiology and Biostatistics and there were monetary constraints. Furthermore, the questions asked about "h/o high BP", "past h/o gestational diabetes", "h/o high cholesterol" and "past h/o depression" were subjectively asked and could not be verified with lab investigations.

## Conclusions

Our study concludes that depression is significantly associated with newly diagnosed type 2 diabetes. Therefore, patients, clinicians and psychiatrists should be informed and educated about the associated burden of depression with newly diagnosed type 2 diabetes. All diabetics within 25 to 60 years should be simultaneously screened for depression. Concomitant preventive strategies for gestational diabetes, nuclear family and high BMI should also be made to prevent mortality/morbidity in 25 to 60 years.

## Abbreviations

OPD: out patient department; BMI: body mass index; SAS: Statistical Analysis Software; h/o: history of; BP: blood pressure: mOR: matched odds ratio; C.I.: confidence interval.

## Competing interests

The authors declare that they have no competing interests.

## Authors' contributions

SP, MSO, MNS, GR and JH participated in conceptualization, study design and protocol preparation. SP performed data collection, its supervision and monitoring. SP and JH did data analysis and its interpretation. SP, MSO, MNS, JH and GR contributed in manuscript drafting and revising. All authors read and approved the final manuscript.
